# Hepatitis B Virus Screening Before Cancer Chemotherapy in Taiwan: A Nationwide Population-Based Study

**DOI:** 10.3389/fmed.2021.657109

**Published:** 2021-07-15

**Authors:** Wei-Chih Sun, Pei-Ling Tang, Wen-Chi Chen, Feng-Woei Tsay, Huay-Min Wang, Tzung-Jiun Tsai, Sung-Shuo Kao, Jin-Shiung Cheng, Wei-Lun Tsai

**Affiliations:** ^1^Division of Gastroenterology and Hepatology, Department of Internal Medicine, Kaohsiung Veterans General Hospital, Kaohsiung, Taiwan; ^2^School of Medicine, National Yang-Ming University, Taipei, Taiwan; ^3^Research Center of Medical Informatics, Kaohsiung Veterans General Hospital, Kaohsiung, Taiwan; ^4^College of Nursing, Kaohsiung Medical University, Kaohsiung, Taiwan; ^5^Department of Nursing, Meiho University, Pingtung, Taiwan; ^6^Department of Medical Education and Research, Kaohsiung Veterans General Hospital, Kaohsiung, Taiwan

**Keywords:** hepatitis, hepatitis B virus, HBV screening, cancer, chemotherapy

## Abstract

**Background:** Reactivation of the hepatitis B virus (HBV) during cancer chemotherapy is a severe and sometimes fatal complication. In 2009, the National Health Insurance (NHI) in Taiwan recommended and reimbursed screening for HBV infection and prophylactic antiviral therapy before cancer chemotherapy. In this study, we determined the HBV screening rate in patients with cancer undergoing chemotherapy in Taiwan.

**Methods:** We retrospectively collected data from the National Health Insurance Research Database on patients who received systemic chemotherapy for solid or hematologic cancers from January 2000 through December 2012. We defined HBV screening based on testing for serum HBsAg within 2 years of the first chemotherapy commencement. We calculated overall and annual HBV screening rates in all patients and subgroups of age, gender, cancer type, hospital level, physician's department, and implementation of NHI reimbursement for HBV screening before cancer chemotherapy.

**Results:** We enrolled 379,639 patients. The overall HBV screening rate was 45.9%. The screening rates were higher in males, those with hematological cancer, those at non-medical centers and medical departments. The HBV screening rates before (2000–2008) and after the implementation of NHI reimbursement (2009–2012) were 38.1 and 57.5%, respectively (*p* < 0.0001). The most common practice pattern of HBV screening was only HBsAg (64.6%) followed by HBsAg/HBsAb (22.1%), and HBsAg/HBcAb/HBsAb (0.7%) (*p* < 0.0001). The annual HBV screening rate increased from 31.5 to 66.3% (*p* < 0.0001). The screening rates of solid and hematological cancers significantly increased by year; however, the trend was greater in solid cancer than in hematological cancer (35.9 and 26.2%, *p* < 0.0001).

**Conclusions:** The HBV screening rate before cancer chemotherapy was fair but increased over time. These figures improved after implementing a government-based strategy; however, a mandatory hospital-based strategy might improve awareness of HBV screening and starting prophylactic antiviral therapy before cancer chemotherapy.

## Introduction

Hepatitis B virus (HBV) is a global health problem. There are approximately two billion people worldwide affected by HBV. According to the 2017 global hepatitis report of the World Health Organization, an estimated 257 million people have chronic HBV infection, which can lead to significant morbidity from cirrhosis and hepatocellular carcinoma (1). Patients with HBV infection are at risk of reactivation when receiving immunosuppressive therapies for various diseases that disturb the host immune system and lead to the reactivation of HBV replication (2). In patients with cancer undergoing chemotherapy, HBV reactivation's incidences were 14–89% for hematological cancer and 4–68% for solid cancer, depending on the chemotherapy regimen and HBV serologic status (3–6). HBV reactivation can result in severe hepatitis, hepatic failure, or death (2, 3, 7). Cancer outcomes may be compromised if cancer treatment is delayed or interrupted by reactivated hepatitis (8). Fortunately, prophylaxis with antiviral agents before chemotherapy reduces HBV reactivation and prevents fatal events (9–11). However, clinicians cannot initiate prophylaxis until the identification of HBV infection. Although there are debates about the best screening pattern, most agencies agree on the importance of HBV screening before chemotherapy. These agencies include the US Centers for Disease Control and Prevention (12), American Association for the Study of Liver Disease (13), European Association for the Study of the Liver (14), and Asian Pacific Association for the Study of the Liver (15). Only the American Society of Clinical Oncology suggests HBV screening for patients who receive specific highly cytotoxic or immunosuppressive therapies such as stem cell transplants or treatment with rituximab (16). Surprisingly, the HBV screening rates before chemotherapy were very low in non-HBV-endemic countries.

The reported screening rates were 16% at the Mayo Clinic (17), 17% at the MD Anderson Cancer Center in the US (18), and 14–31% in Canada (19). Even in HBV-endemic countries such as China, only 17% of patients at the West China Hospital receive HBV screening before chemotherapy (20). In Taiwan, a high endemic region of HBV infection (21), we followed HBV management guidelines published in 2003 (22) and updated in 2008 (23) by the working party of the Asian Pacific Association for the Study of the Liver. The 2003 guidelines recommend that patients who are HBsAg positive undertaking cancer chemotherapy need close monitoring for reactivation and promptly start antiviral therapy before decompensation develops (22). The 2008 guidelines further recommend HBsAg screening before cancer chemotherapy, with the administration of prophylactic antiviral therapy if HBsAg-positive; since 2009, the National Health Insurance (NHI) reimbursed the costs (23). Nevertheless, it has not yet been clarified whether proper HBV screening before cancer chemotherapy is performed in accordance with the NHI guideline. Therefore, we determined the status of HBV screening in patients undergoing cancer chemotherapy in Taiwan.

## Methods

### Data Source

We conducted this retrospective study using the Taiwan National Health Insurance Research Database (NHIRD). Since 1995 in Taiwan, the NHI Program financed the healthcare of more than 99% of residents. The NHIRD includes detailed information from patients' medical records admitted to hospitals; these data include age, sex, diagnosis, interventions, medications, and survival data. This study was approved by the Ethics Committee and the institutional Review Board of the Kaohsiung Veterans General Hospital (VGHKS15-EM10-02). This was a retrospective study using NHIRD that comprises de-identified data, so informed consent was waived. Our hospital's Human Research Committee approved our use of the NHIRD data.

### Definition of Cancer Population and HBV Screening

From January 1, 2000, through December 31, 2012, prospective participants were the 386,390 newly diagnosed patients with cancer (ICD-9-CM 140–195, 200–208) who received their first systemic chemotherapy, including intraarterial, intravenous, or oral chemotherapy. We excluded patients with undetermined sex or younger than 18 years old, leaving 379,639 unique patients. We identified HBV screening based on testing for hepatitis B surface antigen (HBsAg) for any reason within 2 years before chemotherapy initiation. We recorded testing for hepatitis B core antibody (anti-HBc) and hepatitis B surface antibody (anti-HBs) to investigate the practice patterns of HBV screening.

### Outcome Measures and Statistical Analyses

We calculated HBV screening rates in the entire population of 379,639 patients. In subgroup analyses, we compared HBV screening rates using the chi-squared test for the following categories: age, gender; cancer type (solid cancer vs. hematological cancer); hospital level (medical center vs. non-medical center); and physician's department (medical department vs. surgical department). In Taiwan, the medical center has to qualify legal requirement in medical institutional setting, can provide 25 divisions of medical services with more than 25 psychiatric acute beds, and should acquire two qualifications including “Emergency responsible hospital in severely level” and “Cancer-care Quality Certification Program.” We evaluated practice patterns of HBV screening using analysis of variance for subgroup analyses. For patients receiving chemotherapy before (2000–2008) vs. after (2009–2012) implementing Taiwan NHI reimbursement since 2009, we compared overall and subgrouping HBV screening rates using the chi-squared test. We used the Cochran–Armitage test to calculate trends in HBV screening rates by years. To identify factors associated with HBV screening, we used a logistic regression analysis. The predictive factors included age, sex, cancer types, physician's departments, and Taiwan NHI reimbursement implementation. We used the SPSS software (version 10.1; Chicago, IL, USA) for all statistical calculations. A two-sided *p* < 0.05 was statistically significant.

## Results

Table 1 displays the demographic data of all 379,639 patients, including 203,382 (53.6%) males and 176,257 (46.4%) females. The mean age of all patients was 58.4 years. Solid cancers and hematological cancers accounted for 355,741 (93.7%) and 23,898 (6.3%) patients, respectively. There were 251,192 (66.2%) patients from medical centers and 128,447 (33.8%) patients from non-medical centers. The number and proportion of patients treated at medical, surgical, and other departments were 231,678 (61.0%), 145,748 (38.4%), and 2,213 (0.6%), respectively. A total of 227,914 (60.0%) and 151,725 (40.0%) patients initiated cancer chemotherapy before (2000–2008) and after (2009–2012) the implementation of Taiwan NHI reimbursement for HBV screening and prophylactic antiviral therapy in patients with cancer undergoing chemotherapy.

**Table 1:** Demographic data of all patients (Total *N* = 379,639).

	**Number of patients (%)**
**Age** (mean ± S.D.)	58.4 ± 13.5
<35 years	15,900 (4.2%)
35–69 years	278,812 (73.4%)
≧70 years	84,927 (22.4%)
**Gender**	
Female	176,257 (46.4%)
Male	203,382 (53.6%)
**Cancer type**	
Hematological cancer	23,898 (6.3%)
Solid cancer	355,741 (93.7%)
**Hospital level**	
Medical center	251,192 (66.2%)
Non-medical center	128,447 (33.8%)
**Physician's department**	
Medical department	231,678 (61.0%)
Surgical department	145,748 (38.4%)
Other	2,213 (0.6%)
**Era before and after the implementation of NHI reimbursement for HBV screening and prophylactic antiviral therapy before cancer chemotherapy**	
Before: 2000–2008	227,914 (60.0%)
After: 2009–2012	151,725 (40.0%)

Table 2 displays the HBV screening rates in all patients and subgroup analyses. The overall screening rate was 45.9% (174,141/379,639). The HBV screening rates were significantly higher in males than in females (48.6 vs. 42.7%, *p* < 0.0001), in hematological than solid cancers (79.1 and 43.6%, *p* < 0.0001), in patients from non-medical than from medical centers (49.2 and 44.2%, *p* < 0.0001), in patients treated in medical than in surgical departments (55.4 and 30.9%, *p* < 0.0001), and in the era after (2009–2012) than before (2000–2008) the Taiwan NHI reimbursement for HBV screening and prophylactic antiviral therapy in patients with cancer undergoing chemotherapy (57.5 and 38.1%, respectively, *p* < 0.0001).

**Table 2:** HBV screening rates in whole patients and subgroup analyses.

	**Number of patients**	**%**	***p*-value**
**Overall**	174,141	45.9% (174,141/379,639)	
**Age**			<0.0001
<35 years	8,572	53.9% (8,572/15,900)	
35–69 years	131,454	47.1% (131,454/278,812)	
≧70 years	34,115	40.2% (34,115/84,927)	
**Gender**			<0.0001
Female	75,234	42.7% (75,234/176,257)	
Male	98,907	48.6% (98,907/203,382)	
**Cancer type**			<0.0001
Hematological cancer	18,913	79.1% (18,913/23,898)	
Solid cancer	155,228	43.6% (155,228/355,741)	
**Hospital level**			<0.0001
Medical center	110,955	44.2% (110,955/251,192)	
Non-medical center	63,186	49.2% (63,186/128,447)	
**Physician's department**			<0.0001
Medical department	128,153	55.3% (128,153/231,678)	
Surgical department	45,150	30.9% (45,150/145,748)	
**Era before and after the implementation of NHI reimbursement for HBV screening and prophylactic antiviral therapy before cancer chemotherapy**			<0.0001
Before: 2000–2008	86,902	38.1% (86,902/227,914)	
After: 2009–2012	87,239	57.5% (87,239/151,725)	

Table 3 displays the practice patterns of HBV screening before cancer chemotherapy. For all patients, 64.6% (*n* = 112,428) received testing for only HBsAg before cancer chemotherapy, 22.1% (*n* = 38,404) for HBsAg/HBcAb-IgG, and 0.7% (*n* = 8,187) for HBsAg/HBcAb-IgG/HBsAb (*p* < 0.0001). In subgroup analyses, the most common HBV screening pattern before cancer chemotherapy was testing for only HBsAg followed by HBsAg/HBcAb-IgG and HBsAg/HBcAb-IgG/HBsAb.

**Table 3:** Practice patterns of HBV screening in whole patients and subgroup analyses.

	**HBsAg**	**HBsAg HBcAb-IgG**	**HBsAg HBcAb-IgG HBsAb**	***p*-value**
**Overall**	127,550 (73.2%)	38,404 (22.1%)	8,187 (4.7%)	<0.0001
**Age**				
<35 years	6,092 (71.1%)	2,053 (24.0%)	427 (4.9%%)	<0.0001
35–69 years	96,155 (73.1%)	28,952 (22.1%)	6,347 (4.8%)	<0.0001
≧70 years	25,303 (74.2%)	7,399 (21.7%)	1,413 (4.1%)	<0.0001
**Gender**				
Female	55,686 (74.0%)	15,147 (20.2%)	4,401 (5.8%)	<0.0001
Male	71,864 (72.7%)	23,257 (23.5%)	3,786 (3.8%)	<0.0001
**Cancer type**				
Hematological cancer	13,023 (68.9%)	4,809 (25.4%)	1,081 (5.7%)	<0.0001
Solid cancer	114,527 (73.8%)	33,595 (21.6%)	7,106 (4.6%)	<0.0001
**Hospital level**				
Medical center	76,951 (69.4%)	27,823 (25.1%)	6,181 (5.5%)	<0.0001
Non-medical center	50,599 (80.1%)	10,581 (16.7%)	2,006 (3.2%)	<0.0001
**Physician's department**				
Medical department	96,498 (75.3%)	25,893 (20.2%)	5,762 (4.5%)	<0.0001
Surgical department	30,388 (67.3%)	12,338 (27.3%)	2,413 (5.4%)	<0.0001

**Figure 1:**
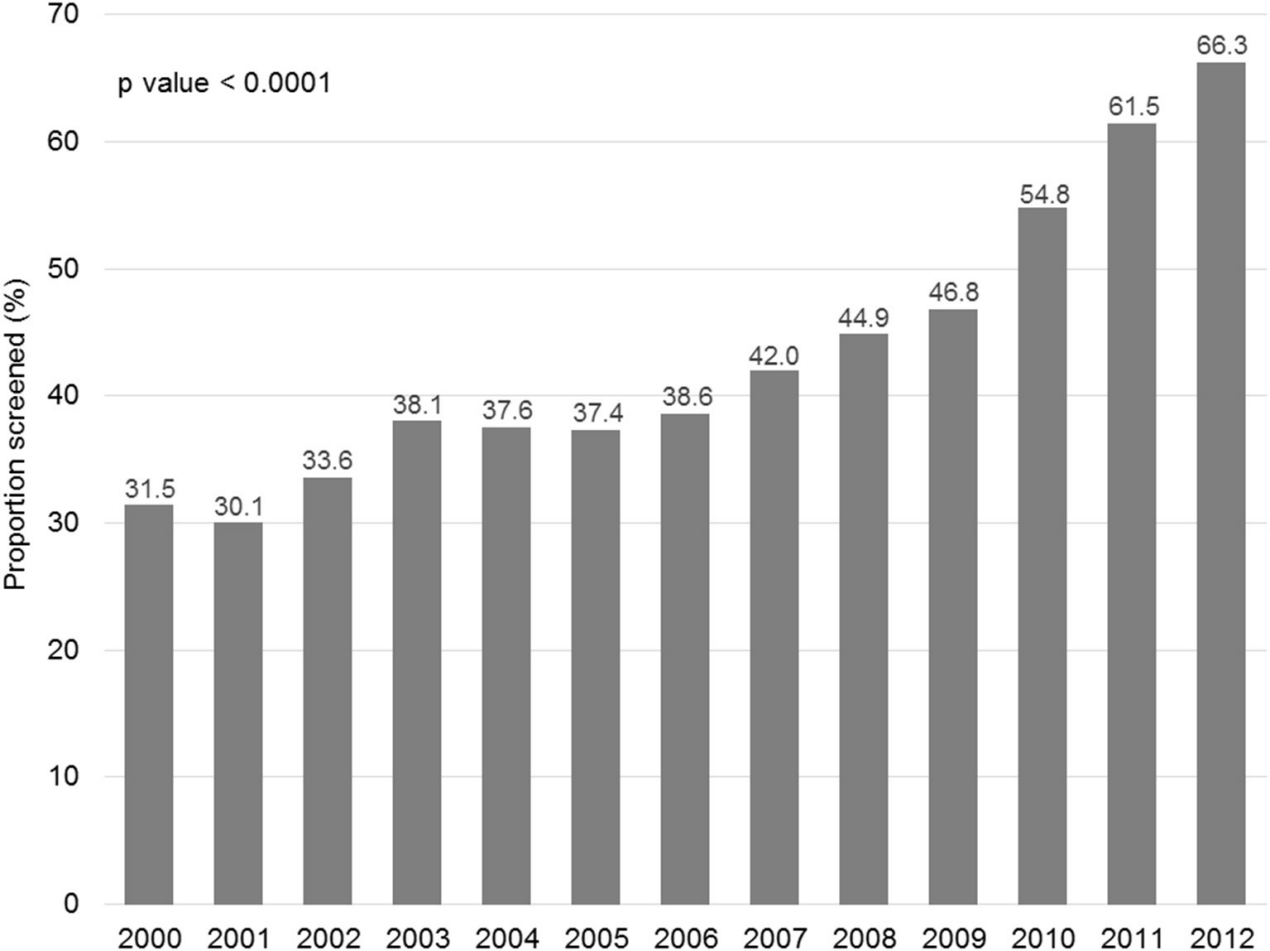
Changes in the annual HBV screening rate.

**Figure 2:**
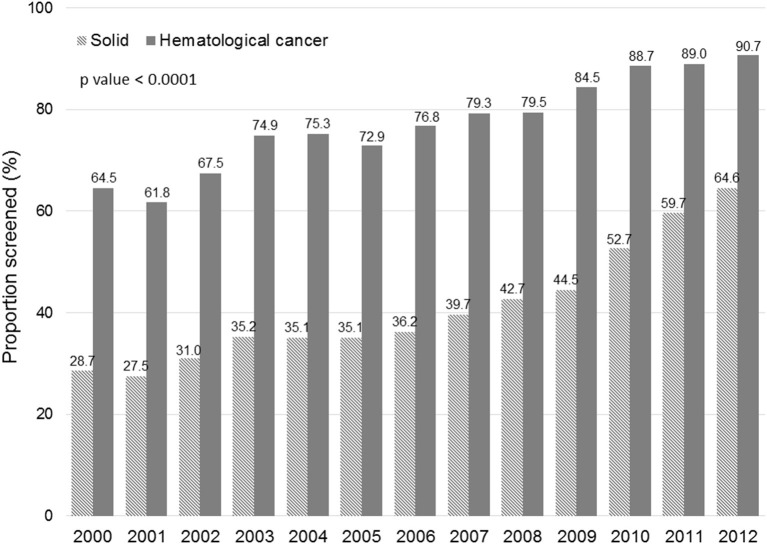
Changes in the annual HBV screening rate in solid and hematological cancers.

Figure 1 displays the evolution of annual HBV screening rates in all patients. These rates increased from 31.5% in 2000 to 66.3% in 2012 (*p* < 0.0001). Figure 2 shows the evolution of annual HBV screening rates in patients with hematological and solid cancers. These rates increased from 64.5 to 90.7% (*p* < 0.0001) in hematological cancers and from 28.7 to 64.6% (*p* < 0.0001) in solid cancers, respectively. Patients with solid cancers had a greater increase in HBV screening (35.9%) than patients with hematological cancers (26.2%) (*p* < 0.0001).

We further investigated the increase in HBV screening rates using a logistic regression analysis. Table 4 displays the multivariate analysis. Factors associated with screening included age (OR = 0.99, *p* < 0.0001), male sex (OR = 1.17, *p* < 0.0001), medical department (OR = 2.64, *p* < 0.0001), hematological cancer (OR = 3.63, *p* < 0.0001), and era (2009–2012) after NHI reimbursement for HBV screening before cancer chemotherapy (OR = 2.46, *p* < 0.0001).

**Table 4:** Factors related to HBV screening before cancer chemotherapy.

	**Screening for HBV (HBsAg)**		
**Clinical factor**	**Odds ratio**	**95% CI**	***p*-value**
Age	0.985	0.985–0.986	<0.0001
Male vs. female	1.166	1.150–1.182	<0.0001
Hematological cancer vs. solid cancer	3.627	3.509–3.749	<0.0001
Medical department vs. surgical department	2.641	2.602–2.680	<0.0001
Era 2 vs. Era 1	2.456	2.422–2.491	<0.0001

* *Era before (Ear 1: 2000–2008) and after (Era 2: 2009–2012) the implementation of NHI reimbursement for HBV screening and prophylactic antiviral therapy before cancer chemotherapy*.

## Discussion

Cancer chemotherapy-induced HBV reactivation is a severe complication because it can cause fatal liver injury (24). Prophylactic anti-HBV therapy before chemotherapy for both hematological and solid cancers can prevent this complication. Effective prophylaxis is available; however, it depends on adequate detection of prior HBV infection. A study showed that early HBV identification correlated with the early use of anti-HBV therapy and reduced the risk of fatal liver failure in patients with HBV receiving cancer chemotherapy (25). If HBV screening does not occur before cancer chemotherapy, strategies to prevent HBV reactivation are meaningless. Recommendations for routine HBV screening before cancer chemotherapy appear in many guidelines; nevertheless, adherence is generally low. In this large cohort study, we measured HBV screening rates before cancer chemotherapy in Taiwan. We found that the overall HBV screening rate was suboptimal (45.9%) but higher than those reported in many other countries (14–34%) (17–22, 26, 27).

The subgroup analyses showed that the overall HBV screening rate was lower in older patients, female patients, those with solid cancers, and those treated in medical centers and surgical departments. The prevalence of HBV increases with age (28); therefore, older patients are more likely to undergo HBV infection testing. Our finding of lower HBV screening rates among older patients might be an underestimate, because we defined HBV screening as testing for serum HBsAg within 2 years of the commencement of first chemotherapy; this constraint may miss patients who underwent testing more than 2 years prior. In Taiwan, males have higher HBsAg carrier rates than females and males are more likely to be tested for HBV infection (29); this finding is similar to our present result. Physicians in medical departments and physicians who have treated hematological cancer tend to know the risk of HBV flares and may be more aware of guidelines regarding HBV screening and prophylactic antiviral therapy before cancer chemotherapy (30). Therefore, our finding of lower HBV screening rates in surgical departments and solid cancer was reasonable. As for lower screening rates in medical centers, physicians in higher-level hospitals might theoretically pay more attention to cancer chemotherapy-related HBV reactivation; therefore, the HBV screening rate should be higher. Kwak reported that HBV screening was less common in community clinics than in teaching hospitals (31). However, our previous study demonstrated low physicians' compliance with HBV screening before cancer chemotherapy in a single medical center (32). A lower hospital level does not necessarily correlate with less knowledge, awareness, or adherence to screening guidelines.

Conversely, physicians who treat larger numbers of patients in medical centers may underestimate the risk of HBV reactivation during cancer chemotherapy and may neglect HBV screening. As for the HBV screening patterns, most patients received testing only for HBsAg, and few underwent universal testing for HBsAg/HBcAb-IgG/HBsAb regardless of the age, gender, cancer type, hospital level, or physician department. This is reasonable because our guidelines recommend only HBsAg testing. The recommendation of our guidelines is different from the ASCO guidelines which suggest testing for HBV by all 3 tests of HBsAg/HBcAb-IgG/HBsAb in patients undergoing highly cytotoxic or immunosuppressive therapies. From the perspective of cost-effectiveness, a recent study found that universal HBV screening before chemotherapy for solid cancer was not cost effective, whereas screening for HBsAg alone was a cost-effective approach (33). However, we did not collect the data to compare the difference in cost-effectiveness of pre-chemotherapy HBV screening between solid and hematological cancers.

Although the overall HBV screening rate was unsatisfactory, it is encouraging that the annual HBV screening rate increased from 31.5% in 2000 to 66.3% in 2012. Both hematological and solid cancers increased annual HBV screening rates; however, the ascending trend was more significant for solid cancers. Our result was different from the results of a survey from the Mayo Clinic (17) and a nationwide survey in Japan (34) that found that HBV screening rates in solid cancer were relatively low and did not increase over time. Potential explanations for a more significant improvement of HBV screening in solid cancers in our study may include (I) that patients with solid cancers had lower baseline annual HBV screening rates so there was larger scope for improvement, and (II) that our strategies to improve HBV screening were more intensive and had a higher impact on patients with solid cancers.

To improve HBV screening before cancer chemotherapy, it is essential to improve awareness among physicians regarding the risk of HBV reactivation during cancer chemotherapy. In a study from St. Michael Hospital in Canada, the screening rate only increased from 14 to 31% after an education intervention (19). In a recent nationwide population-based study from Japan, the screening rate only increased from 51.3 to 67.1% after the Japanese guidelines' announcement (34). In Taiwan, the strategy to improve HBV screening was NHI reimbursement for HBV screening and prophylactic antiviral therapy in patients with cancer receiving chemotherapy, which began in 2009. Our government-based strategy successfully raised the average HBV screening rate from 38.1% in 2000–2008 to 57.5% in 2009–2012. Although the government-based strategy was supposed to be mandatory and legally binding, the annual HBV screening rate only achieved 66.3% in 2012. Some doctors failed to perform HBV screening before cancer chemotherapy due to underestimation of risk of HBV reactivation during cancer chemotherapy, low knowledge regarding the risk for HBV infection, and unawareness of guidelines regarding HBV screening in cancer chemotherapy. Poor compliance with government-based strategy was the main obstacle, and this finding was similar to the results of our previous study. Our hospital developed a novel computer-assisted system that made HBV screening before cancer chemotherapy routine in daily clinical practice to improve poor compliance. In our hospital, the annual screening rate increased from 25.6% in 2009 to 92.3% in 2012. This finding suggests that a hospital-based strategy is also necessary to improve HBV screening in cancer chemotherapy. Just as the findings of a study from the Peter MacCallum Cancer Center in Australia, most physicians (71%) reported that hospital-based policy was the main driver of their practice (35).

Our study's strengths include a large cohort of patients and the provision of strategies to improve the HBV screening rate before cancer chemotherapy in an HBV-endemic area. There were some limitation in our study: (I) the definition of HBV screening before cancer chemotherapy used in our study may overestimate the screening rate; (II) although the screening rate improved based on government-based strategies, we did not know the evolution of prophylactic rate and HBV associated complications; nevertheless, whether improved screening correlates with the commencement of prophylaxis or reduced chemotherapy-induced HBV reactivation is unclear because the NHIRD does not provide more detailed laboratory results to define the outcomes; (III) we did not collect the data of detailed individual chemotherapy agents to show the difference of HBV screening based risk stratification for HBV reactivation; (IV) a nationwide vaccination program since 1983 decreased the prevalence of HBV infection in Taiwan, suggesting the need for further evaluation of the cost-effectiveness of current strategies for HBV screening before cancer chemotherapy.

## Conclusions

Before cancer chemotherapy, the HBV screening rate remained unsatisfactory but gradually increased following the implementation of a government-based strategy. A hospital-based strategy may improve awareness of HBV reactivation during cancer chemotherapy. Policies are necessary to improve HBV screening before cancer chemotherapy.

## Data Availability Statement

The data analyzed in this study is subject to the following licenses/restrictions: Data are available from the National Health Insurance Research Database (NHIRD), which is managed by the National Health Research Institute (NHRI). The raw data cannot be made publicly available. Formal applications should be sent to NHRI and reviewed for approval of data release. Requests to access these datasets should be directed to nhird@nhri.edu.tw.

## Ethics Statement

This study was approved by the Ethics Committee and the institutional Review Board of the Kaohsiung Veterans General Hospital (VGHKS15-EM10-02). This was a retrospective study using NHIRD that comprises de-identified data, so informed consent was waived. Our hospital's Human Research Committee approved our use of the NHIRD data.

## Author Contributions

W-CS, W-CC, F-WT, H-MW, T-JT, S-SK, J-SC, and W-LT contributed to the study concept. W-CS and W-LT design the study. P-LT collected and analyzed the data. W-CS and P-LT performed the statistical analysis. W-CS wrote the draft. W-LT revised the manuscript and gave the final approval for the submission. All authors agreed to the submitted version.

## Conflict of Interest

The authors declare that the research was conducted in the absence of any commercial or financial relationships that could be construed as a potential conflict of interest.
